# The contribution of gut bacteria to insecticide resistance and the life histories of the major malaria vector *Anopheles arabiensis* (Diptera: Culicidae)

**DOI:** 10.1038/s41598-019-45499-z

**Published:** 2019-06-24

**Authors:** Kirsten Barnard, Alexander C. S. N. Jeanrenaud, Basil D. Brooke, Shüné V. Oliver

**Affiliations:** 1 School of Molecular and Cell Biology, Faculty of Science, University of the Witwatersrand, Johannesburg, South Africa; 2 Centre for Emerging Zoonotic and Parasitic Diseases, National Institute for Communicable Diseases of the National Health Laboratory Service, Johannesburg, South Africa; 3 Wits Research Institute for Malaria, MRC Collaborating Centre for Multi-disciplinary Research on Malaria,School of Pathology, Faculty of Health Sciences, University of the Witwatersrand, Johannesburg, South Africa

**Keywords:** Non-model organisms, Evolution

## Abstract

The gut microbiota of mosquitoes is a crucial determinant of their fitness. As such, the biology of the gut microbiota of *Anopheles arabiensis*, a major malaria vector of Southern Africa, was investigated. Two laboratory strains of *An. arabiensis* were used; SENN, an insecticide susceptible strain, and SENN-DDT, a resistant strain. The strains were supplemented with either non-commensal bacteria or antibiotics via a sucrose source to sterilize the gut. The strains were fed the broad-spectrum bactericidal antibiotic gentamicin, or a preferentially gram-positive bactericidal (vancomycin), gram-negative bactericidal (streptomycin) or broad-spectrum bacteriostatic (erythromycin), either by sugar supplementation or by artificially-spiked blood-meal. The effects on adult mosquito longevity and insecticide resistance phenotype were assessed. Bacteria from the midgut of both strains were characterised by MALDI-TOF mass spectroscopy. Bactericidal antibiotics increased longevity in SENN-DDT. Bacterial supplementation increased insecticide tolerance. Antibiotic supplementation via sugar decreased tolerance to the insecticides deltamethrin and malathion. Blood-supplemented vancomycin decreased insecticide resistance, while gentamicin and streptomycin increased resistance. SENN showed a greater gut bacterial diversity than SENN-DDT, with both strains dominated by Gram-negative bacteria. This study suggests a crucial role for bacteria in *An. arabiensis* life history, and that gut microflora play variable roles in insecticide resistant and susceptible mosquitoes.

## Introduction

The immature, aquatic (larval) stages of mosquitoes are challenged by several contaminants^1^. The aquatic phase is also where the larvae obtain the microbes that colonise them and eventually become the commensal gut microbiota^2^. This microbial ecology, mediated by the gut microbiota, is crucial for subsequent survival and success, especially during the adult phase.

It has been demonstrated both directly and indirectly that gut microflora are necessary for the development of larvae. This is primarily because axenic larvae fail to develop. In nature, aquatic larvae obtain colonising gut bacteria from the biofilms that grow around the particulate matter that they feed on^3,4^.

The interplay between microbial flora and adult female fecundity is complex, with antibiotic treatments exerting divergent effects. For example, when the gut of *Anopheles stephensi* was sterilised with tetracycline, significantly fewer eggs were laid^5^. When penicillin and streptomycin were administered to *An. gambiae* via a bloodmeal, fecundity was increased, suggesting an interplay between ingested blood, gut microflora and fecundity^6^.

The role of gut bacteria in adult mosquito longevity, a crucial determinant of vector competence^7^ is less well documented. Currently, the only assessment of the effect of gut bacteria on adult longevity is a study in which various gut-borne bacteria were injected into *An. gambiae*, resulting in reduced longevity^8^.

Gut microflora are a crucial determinant of vector competence, being essential for viral infectivity and protection against *Plasmodium* parasites^9^. Malaria parasite refractoriness appears to be partially mediated by a transient increase in microbial load post blood meal^6^. This increase in bacteria mediates the synthesis and integrity of the protective peritrophic matrix^10^. The microbial load then decreases again 24 hours post blood meal^11^.

The gut microbiome has also recently been demonstrated to be a mediator of insecticide resistance^12^. This was first demonstrated in agricultural pests, with insecticide-degrading gut bacteria having been described in numerous species, including *Plutella xylostella*, where they have been well characterised^13,14^. Symbiont mediated resistance was also described in the beanbug *Riptortus pedestris*^15^ Recently, the gut microbiota of wild-caught organophosphate resistant and susceptible *An. albimanus* has been characterised, and an enrichment of insecticide-degrading bacteria was found in resistant strains^12^.

Insecticide resistance is becoming an increasing threat to vector control efforts^16^. This is particularly true of vector populations in sub-Saharan Africa. The control of the major African malaria vector *An. arabiensis* is proving to be particularly challenging due to insecticide resistance and behavioural plasticity^17^. This opportunistic feeder, which displays both endo- and exophily, is less susceptible to control by indoor residual spraying (IRS) and the distribution of long-lasting insecticide treated nets^17,18^. The tendency of portions of *An. arabiensis* populations to feed and rest outdoors enables them to sustain low-level residual malaria transmission^19^. Controlling outdoor-biting vectors requires novel/additional technologies, and targeting mosquito gut microflora has been suggested^20^. To enable this, the effect of gut microflora on the life history of *An. arabiensis* needs to be assessed amongst other factors. As differences in gut microflora has been demonstrated in insecticide susceptible and resistant strains in *An. albimanus*^12^, the insecticide resistance phenotype must be considered when examining the role of gut microflora on life history. The aim of this study was therefore to assess differential effects of gut microflora on the life histories of insecticide susceptible and resistant laboratory strains of *An. arabiensis*.

## Materials and Methods

Two laboratory strains of *An. arabiensis* were used in this study. The SENN strain was colonised from Sennar in Sudan in 1980, and by continuous DDT selection, the multiple-resistant SENN-DDT strain was developed. SENN-DDT is resistant to DDT, permethrin, deltamethrin, λ-cyhalothrin and malathion. Production of these resistance phenotypes is based on increased metabolic detoxification by specific members of the cytochrome P450, Glutathione S-transferase and general esterase enzyme classes, as well as near-fixation of the L1014F knockdown resistance (*kdr*) mutation^21,22^. Both strains were reared under standard insectary conditions^23^.

### The effect of antibiotic supplementation on *An. arabiensis* adult longevity

Four antibiotics were used to assess the effect of gut bacteria on longevity: The broad-spectrum bactericidal antibiotic gentamycin, the gram-positive narrow-spectrum antibiotic vancomycin, the gram-negative narrow-spectrum antibiotic streptomycin and the broad-spectrum bacteriostatic antibiotic erythromycin. Each was supplemented with 10% sucrose to a final concentration of 2.5 µg/ml. Each antibiotic-treated sucrose solution was supplemented with the preservative methyl paraben to a final concentration of 0.2% to prevent fungal contamination. Each sucrose treatment was delivered to 30 newly-emerged females and 30 newly-emerged males of both the SENN and SENN-DDT strains. Mortality was recorded, with cadavers removed daily. Sucrose treatments were changed twice a week. Adults supplied with a methyl paraben sucrose solution served as controls for each treatment. Mortality was observed until all individuals were dead. This experiment was replicated three times from three cohorts arising from different egg batches. A Kaplan-Meier estimator was used to assess longevity, with a log rank test used as a test for significance.

### The role of gut bacteria on insecticide sensitivity

The role of gut bacteria in the expression of insecticide resistance was examined using two methods. The first was by feeding newly-emerged SENN and SENN-DDT adults with vancomycin-, streptomycin-, gentamicin- and erythromycin-supplemented sucrose only^24^. Untreated sucrose served as a control for these experiments. The second method was by supplementing 10% sucrose with a representative non-commensal gram-positive bacterial species (*Streptococcus pyrogenes* ATCC 19615) or a representative gram-negative species (*Escherichia coli* ATCC 25922). These bacterial strains were selected as neither species were found occurring in either SENN or SENN-DDT, and because they were native microflora, could potentially stimulate immune interaction if ingested by the mosquitoes. Furthermore, neither of these strains has been implicated in the bacterial degradation of deltamethrin or malathion. This would mean that the response observed would be due to the response on the mosquito rather than the addition of a known bacterial degrading agent. The bacterial stock was grown to mid-log phase and the sucrose was supplemented with bacteria to a final concentration of 0.3% in 15 ml. After three days of feeding, SENN-DDT adults (males: *S. pyrogenes*: n = 1236; *E. coli* n = 1350; females *S. pyrogenes*: n = 1472; *E. coli*: 1413) were exposed to either deltamethrin or malathion using standard WHO bioassay susceptibility tests^25^. Mortality was scored 24 hours post-exposure^25^. Adults fed untreated sucrose constituted the control group. Two exposure controls were used for each experiment; exposure to WHO-supplied pyrethroid or organophosphate solvent control tube^25^ or completely unexposed (environmental control). If the mortality in these control groups exceeded 10% the results were discarded. To confirm that any responses observed by bacterial supplementation were not due to bacterial pesticide degradation, bacterial cultures were prepared, grown to mid log phase as described above and then autoclaved. This constituted a heat-killed treatment, which was delivered to the strains by supplementation as described for the live treatment.

As SENN is susceptible to insecticides, changes to their insecticide tolerances were assessed by determining changes in lethal time (LT50) by CDC bottle bioassay (males: *S. pyrogenes*: n = 1042; *E. coli* n = 1028; females *S. pyrogenes*: n = 1112; *E. coli*: 1206)^26^. Adults fed only untreated sucrose constituted the control group. A solvent control exposure and unexposed adults were used for each experiment. Lethal time (LT50) was calculated using Probit analysis^27^ and changes in mortality were assessed using one-way ANOVA, with a 95% confidence interval. All exposures were replicated four times from four cohorts, each arising from four separate egg batches. If the mortality in these control groups exceeded 10% the results were discarded.

### The effect of antibiotic supplementation through blood on the insecticide resistance phenotype

Three day old non-blood fed SENN-DDT females were deprived of sugar for 4 hours prior to allowing them to feed on a Hemotek™ feeder supplemented with 2.5 µg/ml gentamicin, vancomycin, streptomycin or untreated blood, with non-blood fed females serving as a control. The females were allowed to digest blood for 4 hours, after which half the cohort were exposed to either deltamethrin (n = 687) or malathion (n = 624) by WHO bioassay. Control treatments were as previously described (deltamethrin: n = 620; malathion: n = 604). Mortality was scored 24 hours post-exposure. Twenty four hours post blood feed, the initially blood-fed but unexposed cohort was exposed in the same manner as the four hour group (deltamethrin n = 609; malathion n = 612). This experiment was replicated four times from four separate cohorts arising from separate egg batches. This experiment was also replicated using the susceptible SENN strain, with changes in insecticide tolerance assessed by bottle bioassays as described for the sugar-antibiotic treatments described earlier. Changes in mortality were assessed by 1-way ANOVA with a 95% confidence interval.

### Characterisation of the detoxification enzyme activity after antibiotic and bacterial treatment

Newly emerged SENN and SENN-DDT adults were collected, and provided with one of the following treatments; 10% sucrose, gentamicin, vancomycin, streptomycin, live *S. pyrogenes*, Live *E. coli*, heat-killed *S. pyrogenes* or heat killed *E. coli*-supplemented sucrose. The mosquitoes were allowed to feed on the treatments for three days before being cold-killed. 48 individuals of each treatment per sex and strain (e.g. 48 control SENN males, 48 control SENN females etc.) were homogenised in PCR grade water and assessed for cytochrome P450, Glutathione S-transferase and general esterase activity using calorimetric methods^28^.

### Characterisation of gut bacteria of insecticide resistant and susceptible *An. arabiensis*

Fourth instar SENN and SENN-DDT larvae as well as three-day old non-blood fed adults were dissected in sterile phosphate buffered saline to remove the midgut. The midguts of 25 mosquitoes of each strain were inoculated into Brain Heart Infusion broth (Sigma Aldrich: 53286) and incubated for 16 hours at 37 °C. After the incubation, 20 µl of the inoculum was streaked onto either McConkey agar, blood agar or chocolate agar and was incubated at 37 °C for 16 hours. Individual colonies were selected for Matrix-assisted laser desorption time of flight (MALDI TOF). Individual colonies were selected for MALDI TOF mass spectrometry analysis based on colony morphology. Specimens were analysed with a MALDI Biotyper System with a benchtop microflex LT/SH mass spectrometer. Results were analysed using MALDI biotyper 4.0 system software^29^.

### Ethics approval

Ethical approval for this study is stated in the ethical clearance statement from the University of the Witwatersrand, certificate M130534.

## Results

### Antibiotic supplementation and longevity

**Figure 1:**
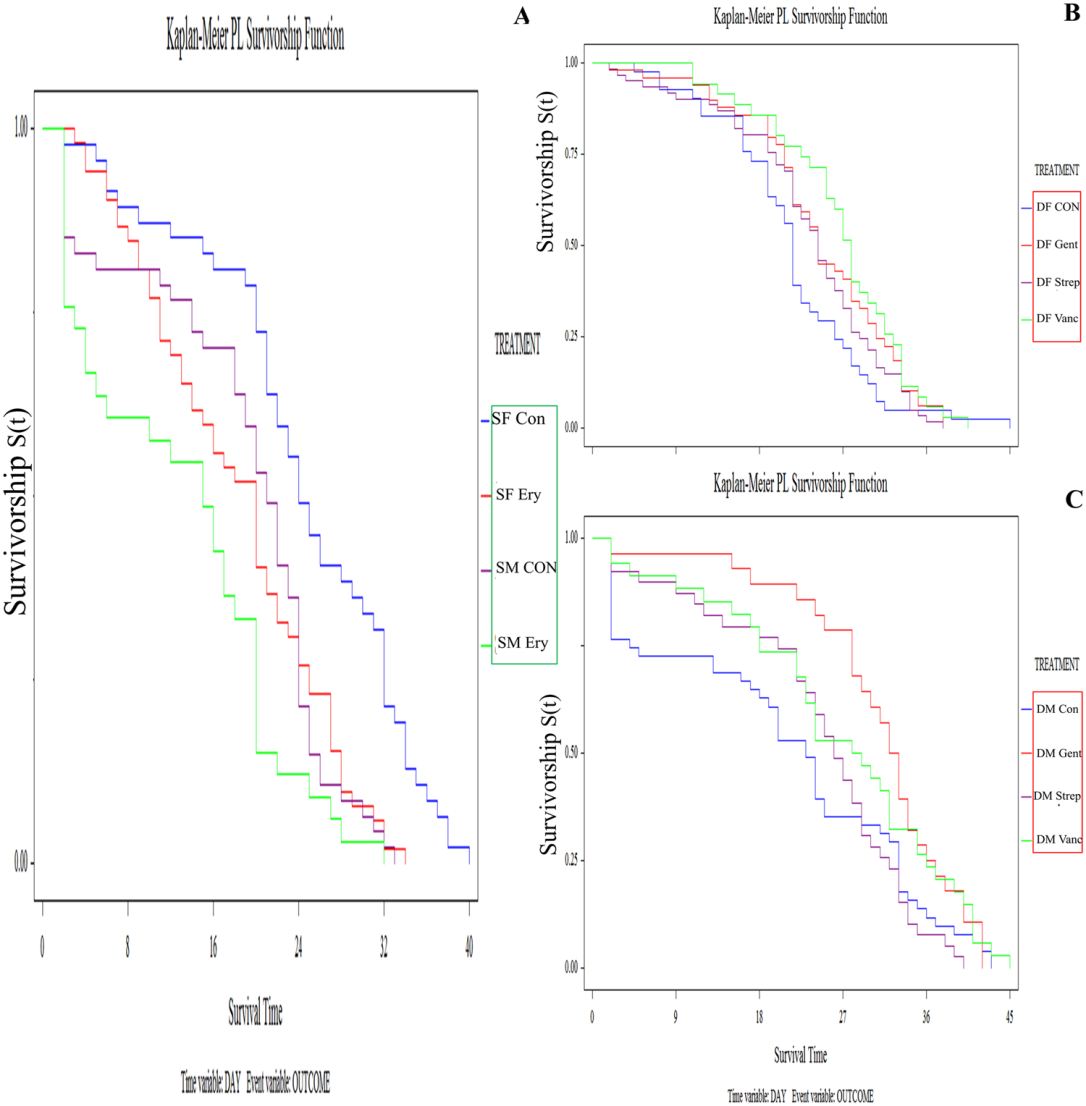
The effect of antibiotic supplementation on *Anopheles arabiensis* longevity. (**A**) For the insecticide-susceptible *Anopheles arabiensis* SENN strain control treatments (SFCon-dark blue and SMCon-purple) lived significantly longer than erythromycin-treated individuals (SFEry-red and SMEry-green). (**B**) Untreated control females of the *Anopheles arabiensis* insecticide-resistant SENN-DDT strain (DFCon-dark blue) lived for a significantly shorter time than gentamicin- (DFGent-red), vancomycin- (DFVan-green), or streptomycin-treated individuals (DFStrep-purple). Vancomycin treated females lived the longest. (**C**) Untreated control males of the *Anopheles arabiensis* insecticide-resistant SENN-DDT strain (DMCon-dark blue) lived for a significantly shorter time than gentamicin- (DMGent-red), vancomycin- (DMVan-green), or streptomycin-treated individuals (DMStrep-purple). Gentamicin-treated males lived the longest. Significance was determined by the Log-Rank test. Insecticide susceptible strains are denoted by a green block and insecticide resistant strains are denoted by a red block.

SENN and SENN-DDT adult longevitieswere differentially affected by selective antibiotic treatment. In SENN, bactericidal antibiotics did not affect longevity in females (Log rank test: *P* = 0.19, df = 3, χ^2^ = 4.73) or males (Log rank test: *P* = 0.14, df = 3, χ^2^ = 5.51). The broad-spectrum bacteriostatic erythromycin did however reduce the longevity of SENN females (Log rank test: *P* < 0.01, df = 1, χ^2^ = 12.01) and SENN males (Log rank test: *P* = 0.02, df = 1, χ^2^ = 5.09) (Fig. 1A).

By contrast, bactericidal antibiotic treatments significantly affected adult longevities in the SENN-DDT strain. Gentamicin, streptomycin and vancomycin increased longevity in SENN-DDT females (Log rank test: *P* = 0.01, df = 3, χ^2^ = 10.73) (Fig. 1B) and males (Log rank test: *P* = 0.03, df = 3, χ^2^ = 8.76) (Fig. 1C). Vancomycin treatment was associated with the greatest longevity in SENN-DDT females, while Gentamicin resulted in the greatest longevity in SENN-DDT males. The log rank test was used as data was not censored with an α-value of 0.05%

### The contribution of gut bacteria and immune response to the insecticide resistance phenotype

**Figure 2:**
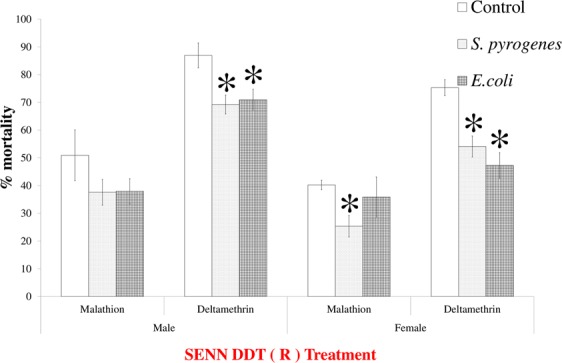
The effect of the supplementation of non-commensal bacteria on the insecticide resistance phenotype of *Anopheles arabiensis* SENN-DDT. When the insecticide resistant SENN-DDT strain was supplemented with either gram-positive (*Streptococcus pyrogenes*) or gram-negative bacteria (*Escherichia coli*), deltamethrin-induced mortality decreased in both males and females. Bacterial supplementation had no effect on male malathion-induced mortality, while only *S. pyrogenes* supplementation significantly reduced malathion-induced mortality in females. Asterisks indicate a significant difference from the untreated control of the same sex and insecticide exposure. The treatment label is in red to indicate insecticide resistance.

Supplementing the diet of SENN-DDT with a representative non-commensal gram-positive bacterium (*S. pyrogenes*) and gram-negative bacterium (*E. coli*) augmented the malathion and deltamethrin resistance phenotype in this strain. Deltamethrin-induced mortality was decreased in females for both *S. pyrogenes* (2-sample t-test: *P* = 0.01, t = 3.36, df = 26) and *E. coli* (2-sample t-test: *P* < 0.01, t = 4.55, df = 18). This was also true for male *S. pyrogenes* supplementation (2-sample t-test: *P* < 0.01, t = 3.79, df = 13) as well as *E. coli* supplementation (2-sample t-test: p *P* = 0.01, t = 3.23 df = 13.6). The results were less marked for malathion. Only supplementation with *S. pyrogenes* significantly decreased malathion-induced mortality (2-sample t-test: *P* = 0.02, t = 2.77, df = 9), while *E. coli* supplementation did not affect malathion-induced mortality in females (2-sample t-test: *P* = 0.60, t = 0.58, df = 6). For SENN-DDT males, neither *S. pyrogenes* (2-sample t-test: *P* = 0.31, t = 1.15, df = 4.2) nor *E. coli* (2-sample t-test: *P* = 0.16, t = 1.53, df = 10) affected malathion-induced mortality (Fig. 2).

**Figure 3:**
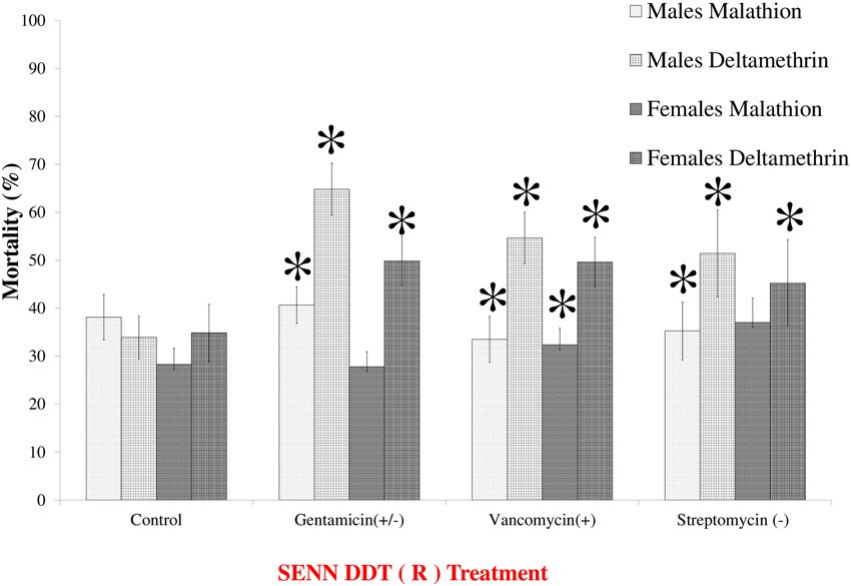
The effect of antibiotic supplementation on the insecticide resistance phenotype of *Anopheles arabiensis* SENN-DDT. Vancomycin treatment significantly increased malathion an deltamethrin-induced mortality in males and females. Gentamicin increased deltamethrin-induced mortality in females. Streptomycin increased malathion and deltamethrin-induced mortality in males and deltamethrin-induced mortality in females. Asterisks denote a significant change from untreated controls of the same sex of the same sex and insecticide exposure. The treatment label is in red to indicate insecticide resistance.

Antibiotic treatment generally increased insecticide-induced mortalities, with deltamethrin resistance being the most affected. All treatments increased deltamethrin-induced mortality in females (2-sample t-test: gentamicin- *P* = 0.02, t = −2.51, df = 25.1; vancomycin: *P* < 0.01, t = −3.54, df = 24.8; streptomycin- *P* < 0.01, t = −3.19, df = 23.6). This was true for males as well (2-sample t-test: gentamicin- *P* < 0.01, t = −3.42, df = 34.6; vancomycin: *P* < 0.01, t = −4.19, df = 33.8; streptomycin- *P* < 0.01, t = −3.52, df = 34.5). For malathion-induced mortality in females, only vancomycin significantly increased mortality (2-sample t-test: *P* < 0.01, t = −3.54, df = 27.2), while gentamicin and streptomycin did not (2-samples t-test: gentamicin- *P* = 0.22, t = −1.24, df = 31.4; streptomycin- *P* = 0.09, t = −1.76, df = 31.9). Similarly for males, malathion-induced mortality was increased by vancomycin treatment (2-sample t-test: *P* = 0.02, t = −2.53, df = 34.9), but not treatments with gentamicin or streptomycin (2-samples t-test: gentamicin- *P* = 0.06, t = −1.92, df = 31.2; streptomycin- *P* = 0.36, t = −0.94, df = 32) (Fig. 3). Living and heat-killed bacteria elicited the same responses - mortalities did not differ significantly when supplied with either treatment (Table 1).

**Table 1:** Insecticide-induced mortality post bacterial treatment in SENN-DDT after supplementation with live or heat-killed bacteria.

		Deltamethrin				Malathion			
		Male	P (T)	Female	P (T)	Male	P (T)	Female	P (T)
*S.pyrogenes*	Live	55.8 (±3.87)	0.34 (−0.95)	52.8(±3.46)	0.99 (−0.01)	68.9 (±2.97)	0.39(−0.86)	25.3(±3.86)	0.48 (−0.72)
	Dead	64.6 (±2.93)		52.6(±4.06)		73.5 (±3.94)		30.3(±3.63)	
*E. coli*	Live	80.7 (±3.85)	0.06 (2.04)	55.5(±3.87)	0.15 (−1.48)	37.6 (±4.64)	0.73 (0.35)	30.1(±3.46)	0.12 (1.65)
	Dead	66.7 (±5.63)		64.7(±2.93)		35.5 (±3.82)		21.8(±2.11)	

P-values were determined by 2-sample t-test at 95% confidence intervals.

**Figure 4:**
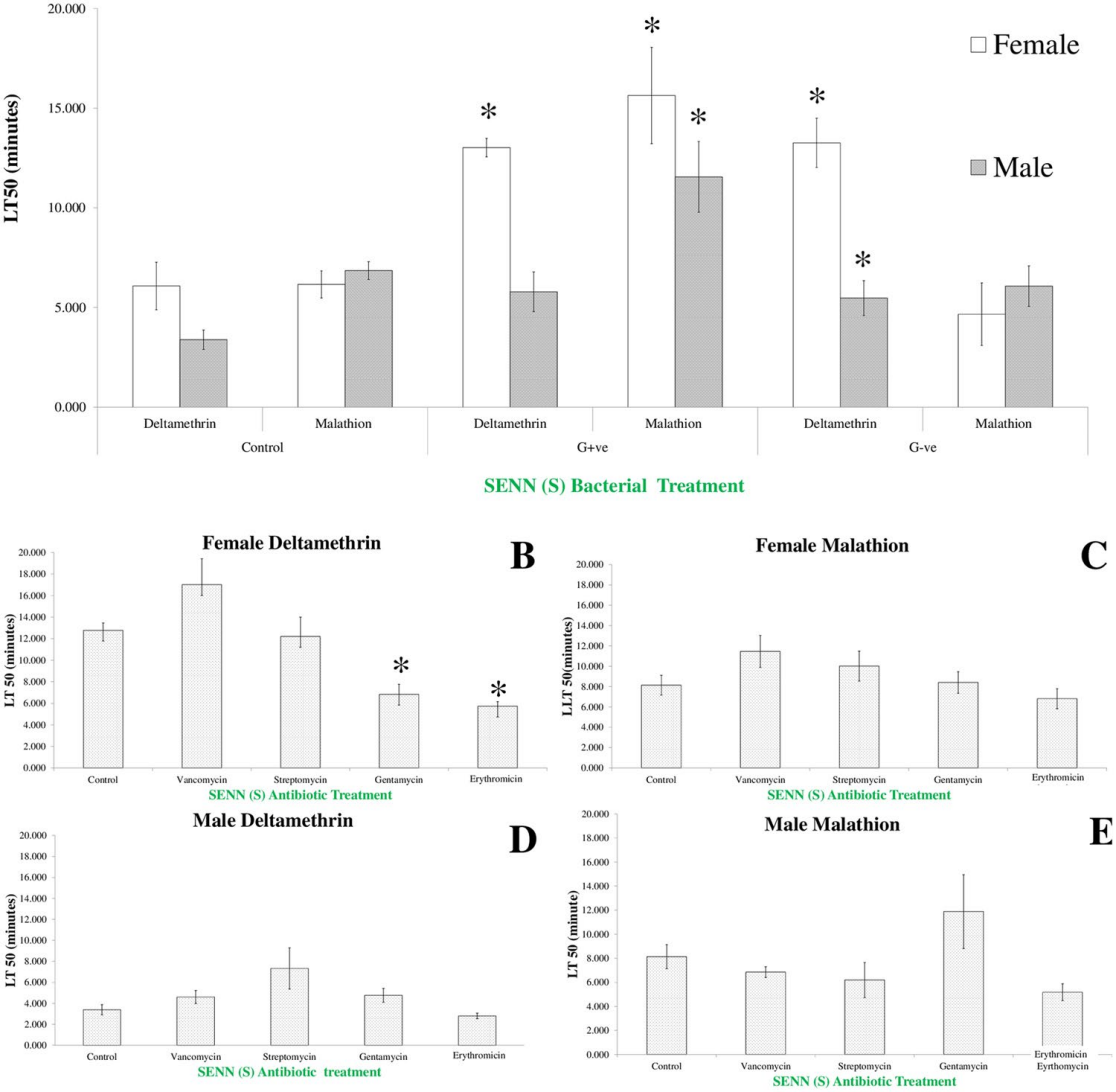
The effect of bacterial and antibiotic supplementation on insecticide-induced LT50 (time to 50% mortality) values in *Anopheles arabiensis* SENN. (**A**) Supplementation with gram-positive bacteria (*Streptococcus pyrogenes*) increased deltamethrin and malathion LT50s in females, but only malathion LT50 in males. Gram-negative (*Escherichia coli*) treatment increased male and female deltamethrin LT50, but had no effect on malathion lethal time. Asterisks indicate a significant difference from the exposed, untreated control of the same sex and insecticide exposure. (**B**) The effect of antibiotic-supplemented sugar on female deltamethrin LT50. Asterisks indicate a significant difference from the exposed, untreated control. (**C**) The effect of antibiotic-supplemented sugar on female malathion LT50. (**D**) The effect of antibiotic-supplemented sugar on male deltamethrin LT50. (**E**) The effect of antibiotic-supplemented sugar male malathion LT50. The treatment label is in green to indicate insecticide susceptibility.

To determine whether similar effects could be observed in the insecticide susceptible SENN strain, LT50s were determined for malathion and deltamethrin. When supplemented with bacteria, deltamethrin lethal time in females was increased after both gram-positive treatment (2-sample t-test: *P* = 0.01, df = 11.2, t = −3.34) and gram-negative treatment (2-sample t-test: *P* = 0.03, df = 8.6, t = −2.26). Female malathion lethal time was increased after gram-positive treatment (2-sample t-test: *P* < 0.01, df = 12, t = −5.24) but not gram-negative treatment (2-sample t-test: *P* = 0.42, df = 4.2, t = 0.88). In males, deltamethrin resistance was not affected by gram-positive treatment (2-sample t-test: *P* = 0.09, df = 4.4, t = −2.17), but was significantly increased by gram-negative treatment (2-sample t-test: *P* < 0.01, df = −7.72, t = 3.9). Malathion LT50 in males was significantly increased after gram-positive treatment (2-sample t-test: *P* = 0.04, df = 6, t = −2.56) but not gram-negative treatment (2-sample t-test: *P* = 0.52, df = 4.1, t = 0.7). For females, there was no significant difference in the deltamethrin lethal time induced by gram-positive or negative treatment (2-sample t-test: *P* = 0.87, df = 3.8, t = 0.18) but gram-positive treatment resulted in a significantly higher malathion lethal time than gram-negative treatment (2-sample t-test: *P* = 0.01, df = 5.2, t = −3.81). For males, gram-negative treatment resulted in a significantly higher deltamethrin lethal time than gram-positive treatment (2-sample t-test: *P* < 0.01, df = 5.7, t = 4.7). For malathion, gram-positive treatment resulted in a significantly higher lethal time than gram-negative treatment (2-sample t-test: *P* = 0.04, df = 4.8, t = −2.67) (Fig. 4A).

Antibiotic treatment had a less marked effect on insecticide tolerance than bacterial supplementation. For deltamethrin, lethal time was significantly reduced by gentamicin (2-sample t-test: *P* < 0.01, df = 4, t = 5.15) and erythromycin treatment (2-sample t-test: *P* < 0.01, df = 5, t = −8.73) in females. No significant changes were observed in males after any antibiotic treatment (1-way ANOVA: F (4, 13) = 2.67, *P* = 0.08). Malathion lethal time was not affected by any treatment in females (1-way ANOVA: F(4, 13) = 2.27 *P* = 0.11) or males (1 way ANOVA: F (4, 13) = 3.17) *P* = 0.11) (Fig. 4B). All mortality data was found to be normally distributed using the Shapiro-Wilks test. An alpha value of 0.05% was used in all cases. Environmental control and solvent control mortality did not exceed 5% for any treatment.

### The effect of antibiotic supplemented blood on the insecticide resistant phenotype

**Figure 5:**
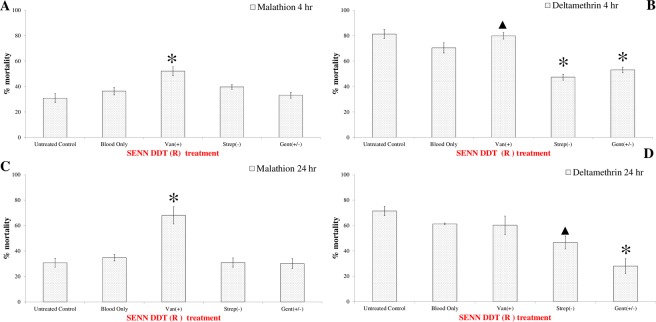
The effect of antibiotic-treated blood on the insecticide resistance phenotype of *Anopheles arabiensis* SENN-DDT. (**A**) Malathion-induced mortality 4 hours post antibiotic supplemented blood-meal. Asterisks indicate a significant difference from the exposed, untreated control. (**B**) Deltamethrin-induced mortality 4 hours post antibiotic supplemented blood-meal. Symbols indicate a significant difference from the exposed, untreated control, with the different symbols indicative a significant difference between treatments. (**C**) Malathion-induced mortality 24 hours post antibiotic supplemented blood-meal. Asterisks indicate a significant difference from the exposed, untreated control. (**D**) Deltamethrin-induced mortality 4 hours post antibiotic supplemented blood-meal. Symbols indicate a significant difference from the exposed, untreated control, with the different symbols indicative a significant difference between treatments. The treatment label is in red to indicate insecticide resistance.

As antibiotics did not have a marked effect on the tolerance of SENN to insecticides, the effect of blood-borne antibiotics was only examined in SENN-DDT. The effect of blood-borne antibiotics differed 4 hours and 24 hours post blood meal. After 4 hours, vancomycin induced the greatest increase in deltamethrin-induced mortality, and resulted in a significantly higher mortality compared to the control or untreated blood (1-way ANOVA: F (2, 66) = 3.47, *P* = 0.03). By contrast, streptomycin and gentamicin treatment resulted in decreased deltamethrin-induced mortality (1-way ANOVA: F (3, 166) = 7.26, *P* < 0.01) after 4 hours. For malathion-induced mortality, only vancomycin treatment resulted in increased mortality (1-way ANOVA: F (2, 48) = 11.4, *P* < 0.01) (Fig. 5A).

After 24 hours post blood meal, the deltamethrin-induced mortality of vancomycin-treated females no longer differed from the control and untreated, blood-fed cohorts (1-way ANOVA: F (2, 44) = 2.09, *P* = 0.13). Deltamethrin-induced mortality in vancomycin-treated females was significantly lower after 24 hours compared to 4 hours (2-sample t-test: *P* = 0.01, df = 30, t = 2.67). Gentamicin and streptomycin treatment still resulted in reduced mortality after 24 hours (1-way ANOVA: F (3, 58) = 19.7, *P* < 0.01). Only vancomycin-treated females had increased malathion-induced mortality after 24 hours (1-way ANOVA: F (2, 32) = 22.4, *P* < 0.01) (Fig. 5B). No significant difference in insecticide tolerance, measured as LT50, was found in any treatment of the SENN strain (1-way ANOVA: F (4, 13) = 2.3, *P* = 0.58) (Supplementary Table 2). All mortality data were found to be normally distributed using the Shapiro-Wilks test. An alpha value of 0.05% was used in all cases. Environmental control and solvent control mortality did not exceed 5% for any treatment.

### Characterisation of the detoxification enzyme activity after antibiotic and bacterial treatment

**Figure 6:**
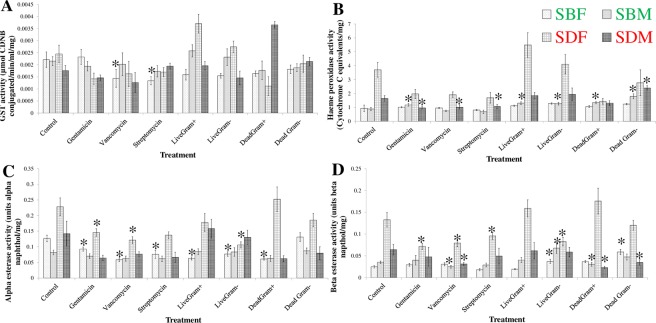
The effect of antibiotic and bacterial-sugar supplementation on the detoxification enzyme activity of *Anopheles arabiensis* SENN and SENN-DDT. (**A**) GST activity in SENN and SENN-DDT. Asterisks indicate a significant difference from the untreated control of the same sex and strain. Vancomycin and Streptomycin reduced GST activity in SENN females, but this was the only significant difference. (**B**) Cytochrome P450 activity in SENN and SENN-DDT. Asterisks indicate a significant difference from the untreated control of the same sex and strain. No consistent pattern was observable in the activity of this class of enzyme. (**C**) Alpha-esterase activity in SENN and SENN-DDT. Asterisks indicate a significant difference from the untreated control of the same sex and strain. Where significant differences were observed, the activity was significantly lower. (**D**) Beta-esterase activity in SENN and SENN-DDT. Asterisks indicate a significant difference from the untreated control of the same sex and strain. For the antibiotic treatment, all the significant changes were significantly lower. For live gram-negative bacterial treatment, the significant changes were significantly higher, except for SENN-DDT females, were the activity was significantly lower. Dead gram-positive bacteria resulted in significantly lower activity where activity was significantly different. Treatment with dead gram-negative bacteria resulted in significantly higher beta esterase activity in SENN females, but significantly lower activity in SENN-DDT males. No consistent patterns in enzyme activity were observed, and the changes in enzyme activity were not congruent with the observed bioassay results.

GST activity remained largely unchanged, with only a reduction in activity in SENN females, after vancomycin treatment (2-sample t-test: *P* = 0.04, df = 22, t = 2.13) and streptomycin (2-sample t-test: *P* = 0.04, df = 19.3, t = 2.23) (Fig. 6A).

Cytochrome P450 was most affected in males. In SENN males, gentamicin treatment increased P450 activity (2-sample t-test: *P* = 0.04, df = 22, t = −2.09), live *S. pyrogenes* increased activity (2-sample t-test: *P* < 0.01, df = 21.9, t = −2.89), live *E. coli* increased activity (2-sample t-test: *P* = 0.02, df = 21.6, t = −2.63), as did heat-killed *S. pyrogenes* (2-sample t-test: *P* < 0.01, df = 21.4, t = −3.27) and heat-killed *E. coli* (2-sample t-test: *P* < 0.01, df = 18.1, t = −4.36). In SENN-DDT males, gentamicin decreased activity (2-sample t-test: *P* < 0.01, df = 22, t = 2.98), as did vancomycin (2-sample t-test: *P* = 0.02, df = 20.3, t = 2.48) and streptomycin (2-sample t-test: *P* = 0.03, df = 20.3, t = 2.28). Heat-killed gram negative bacteria increased activity (2-sample t-test: *P* < 0.01, df = 21.4, t = −2.85) (Fig. 6B).

In contrast, females displayed the most changes in terms of α-esterase activity. SENN females had decreased activity after gentamicin treatment (2-sample t-test: *P* = 0.01, df = 18.7, t = 2.81), vancomycin treatment (2-sample t-test: *P* < 0.01, df = 22, t = 5.81) and streptomycin treatment (2-sample t-test: *P* < 0.01, df = 21, t = 3.11), live *S. pyrogenes* treatment (2-sample t-test: *P* < 0.01, df = 22, t = 5.64), live *E. coli* treatment (2-sample t-test: *P* < 0.01, df = 22, t = 16.11) as well as heat-killed *S. pyrogenes* treatment (2-sample t-test: *P* < 0.01, df = 22, t = 16.11). In SENN-DDT females, gentamicin treatment decreased activity (2-sample t-test: *P* = 0.01, df = 22, t = 2.69), as did vancomycin treatment (2-sample t-test: *P* < 0.01, df = 22, t = 3.35) and streptomycin treatment (2-sample t-test: *P* < 0.01, df = 22, t = 3.00) and live *E. coli* treatment (2-sample t-test: *P* < 0.01, df = 22, t = 4.07) (Fig. 6C).

A variety of changes were observed in β-esterase activity. In SENN females, activity was increased by live *E. coli* treatment (2-sample t-test: *P* = 0.04, df = 22, t = 3.00), heat-killed *S. pyrogenes* treatment (2-sample t-test: *P* < 0.01, df = 22, t = −3.32) as did heat -killed *E. coli* (2-sample t-test: *P* < 0.01, df = 22, t = −5.29). In SENN males, vancomycin treatment decreased activity (2-sample t-test: p = 0.02, df = 21.2, t = 2.46), while live *E. coli* treatment increased activity (2-sample t-test: *P* = 0.01, df = 22, t = −2.58). In SENN-DDT females activity was decreased by gentamicin treatment (2-sample t-test: *P* < 0.01, df = 22, t = 3.36), vancomycin treatment (2-sample t-test: *P* < 0.01, df = 22, t = 2.88) streptomycin treatment (2-sample t-test: *P* = 0.04, df = 22, t = 2.03) as well as live *E. coli* treatment (2-sample t-test: *P* = 0.01, df = 22, t = 2.59). In SENN-DDT males activity was decreased by vancomycin activity (2-sample t-test: *P* = 0.01, df = 22, t = 2.61), heat-killed *S. pyrogenes* treatment (2-sample t-test: *P* < 0.01, df = 22, t = 3.29) as well as heat-killed *E. coli* treatment (2-sample t-test: *P* = 0.04, df = 22, t = 2.14) (Fig. 6D).

### Comparison of gut bacterial composition between insecticide resistant and susceptible *An. arabiensis*

In fourth instar larvae, SENN had eight predominant culturable aerobic bacteria, compared to four in SENN-DDT. *Klebsiella varicolla* was the predominant species in both strains. In the SENN strain, two gram-positive species were detected, *Staphylococcus hominis* and *Corynebacterium amycolatum*, while none were detected in fourth instar SENN-DDT larvae. *Elizabethkingia mengioseptica*, which has been found to be a dominant midgut bacterium in *An. stephensi*, was observed in SENN larvae. Although the number of dominant species in the SENN strain decreased, there were still more species represented in SENN than SENN-DDT. In both strains, the dominant species in larvae were *Klebsiella* spp., while in adults the dominant species were *Enterobacter* spp. The data is summarised in Supplementary Fig. 1.

## Discussion

Recent literature has demonstrated that gut bacteria play a crucial role in the life histories of mosquitoes, with particular relevance to larval development and vector competence^3,30^. Less well examined is the interplay between insecticide resistance phenotypes and gut bacteria. An important factor that has been determined is that insecticide resistant *Anopheles* mosquitoes have lower gut bacterial diversity than their susceptible counterparts^12^. Therefore, antibiotic supplementation, which could alter gut bacterial load and/or diversity, could affect adult longevity.

Longevity in the insecticide susceptible SENN strain was reduced after exposure to the broad-spectrum bacteriostatic erythromycin. For resistant SENN-DDT, bactericidal antibiotic treatment led to increased longevity, with males and females responding differently. While males lived longest when exposed to the broad-spectrum gentamicin, gram-positive vancomycin treatment resulted in greatest longevity in SENN-DDT females. This suggests a sex-linked contribution of gut bacteria to longevity, which also varies between insecticide susceptible and resistant strains. This may possibly be due to the differing proportions of gut bacteria present in the strains. It has been suggested that bacterial effects on larval development may be due to alteration of oxidative conditions in the gut^31^. This may contribute to the difference seen in resistant and susceptible adults, as it has previously been demonstrated that insecticide resistant anophelines differ in their capacity to cope with oxidative stress^32^, and as such alteration of redox state may differentially affect longevity, given that redox state is a major determinant of longevity^33^.

That gut bacteria can affect the expression of insecticide resistant phenotypes is supported by data from this study. Antibiotic treatment of SENN-DDT significantly increased subsequent deltamethrin and malathion induced mortality, but the effect was less marked in SENN. Previous studies have demonstrated that selection for insecticide resistance reduces gut bacterial diversity by enriching insecticide degrading bacteria in the gut^12^. This may explain why the bactericidal antibiotics and not bacteriostatic antibiotics (detailed in Appendix Table 3A,B) had a greater effect on SENN-DDT and not SENN. The differing antibiotics had differing effects on insecticide-induced mortality in SENN-DDT. Vancomycin treatment induced the greatest effect, increasing mortality in all insecticide treatments. Gentamicin and streptomycin did not affect malathion-induced mortality in SENN-DDT females. This may be due to the similarity of the activity of these antibiotics; they are both aminoglycosides that inhibit protein synthesis by interfering at the 30 S ribosomal subunit. Streptomycin’s specific toxicity towards gram-negative bacteria is due to preferential permeation of the gram-negative cell wall^34,35^. These data support a previous study demonstrating that larval exposure to tetracycline can negate temephos resistance in *An. stephensi*^36^.

Supplementation with the gram-positive bacterium *Streptococcus pyrogenes* decreased both malathion and deltamethrin-induced mortality in SENN-DDT females. This pattern was also observed in SENN females. *Escherichia coli* had less of a marked effect on resistance, and had no effect on malathion resistance in either SENN-DDT males or females. Similarly, *E. coli* had no effect on malathion-induced lethal time in either gender of the SENN strain. This highlights three factors. Vancomycin had the greatest effect on longevity and decreasing resistance, while gram-positive supplementation had the greatest effect on female resistance, suggesting a role for gram-positive bacteria in the life history of SENN-DDT, and to an extent, SENN. Secondly, the less marked effect of *E. coli* on resistance may be due to the fact that this bacterium, to date, has never been implicated in malathion degradation. Thirdly, the antibiotic and bacterial treatment had a greater effect on resistance/tolerance to the pyrethroid insecticide deltamethrin than to the organophosphate malathion. Malathion resistance has previous been demonstrated to be less affected by blood treatments^37,38^, and this phenotype was more stable in this strain.

It is possible that the observations made regarding resistance may be due to the treatments (either antibiotic or bacteria) eliciting a change in the activity of xenobiotic detoxifying enzymes. If this hypothesis is correct, then factor reducing resistance (antibiotics) would result in reduced detoxification enzyme activity and the resistance increasing factors would increase detoxification enzyme activity (as seen in^26^ for example). This pattern is not consistently observed. Antibiotic supplementation decreased some cytochrome P450 and general esterase activities, but this was neither consistent nor observed in all strains. Conversely, bacterial supplementation did not consistently result in increased detoxification activity. As previous studies on this strain have demonstrated that deltamethrin and malathion resistance is metabolically mediated^32,38^, this response is not due to target site mutations. The detoxification enzyme data presented in this study can also not account for these changes. As both live and dead bacteria elicit an increase in insecticide tolerance it is suggested that other factors may be involved. A likely candidate is the interplay with the immune system that may alter redox state, which is has been demonstrated to affect insecticide resistance^32^. Another factor may be the alteration of gut microflora. Although the results of this study cannot conclusively implicate either system, it does raise new questions about the biology of insecticide resistance and adds to the body of evidence concerning gut bacteria and insecticide resistance.

Blood and sugar are digested differently in mosquitoes^39^. The choice of delivery system may affect the response to antibiotics. Antibiotics and other blood-borne host factors have been shown to affect mosquito biology^6,40^ and therefore the effect of blood-administered antibiotic was examined. Subsequent exposures to insecticides at four and twenty four hours post blood meal were chosen as these times correspond to the peaks in transcriptional changes and the greatest decrease in insecticide toxicity post blood meal^41–44^. Insecticide-induced mortality four hours post antibiotic-spiked blood feeding provided the closest match to the results obtained by sugar supplementation, with vancomycin increasing malathion and deltamethrin-induced mortalities, and by the greatest amount. Streptomycin and gentamicin decreased deltamethrin-induced mortality. Twenty-four hours post antibiotic-spiked blood feeding, the pattern changed, with vancomycin treated females showing an increased malathion-induced mortality (even exceeding that induced at 4 hours post blood meal), while deltamethrin-induced mortality matched pre-feeding levels. Malathion-induced mortality following streptomycin and gentamicin treatments remained unchanged, with deltamethrin-induced mortality decreased, and gentamicin-induced mortality even lower at 24-hour post blood feeding. Therefore, both sugar and blood administered antibiotic had an effect on insecticide resistance in the SENN-DDT strain. The reduction in resistance caused by vancomycin even negated the reduced insecticide toxicity that a single blood meal has been demonstrated to have^43,45^. As this effect was not due to altered detoxification enzyme activity, it can be suggested that antibiotic-induced changes in bacterial load and/or diversity may play a role.

A preliminary investigation of the predominant culturable gut bacteria of the two *An. arabiensis* strains largely mirrors results from previous studies on anophelines, although these data present the first report of *Elizabethkingia mengioseptica* in the gut flora of this species. In both strains, the dominant larval populations were *Klebsiella* species, specifically *Klebsiella varicolla*. Adult populations were dominated by *Enterobacter asburiae*.

There was a notably reduced bacterial species diversity in the insecticide resistant SENN-DDT strain compared to the susceptible SENN strain (SENN larval diversity index [DI]: 3.39, SENN-DDT DI: 2.60; SENN adult DI: 2.17, SENN-DDT adult DI: 1.78). Not only does SENN-DDT have a lower diversity, but the relative proportions of the common bacterial species differ. An enrichment of *Klebsiella* species was observed in SENN-DDT larvae, as was similarly observed in fenitrothion-resistant *An. albimanus*^12^. Furthermore, species implicated in pesticide degradation make up the greater proportion of gut bacteria in SENN-DDT adults. As with previous studies, gram-negative bacteria predominate^12,29^, which may explain the marked effects of altering gram-positive populations. It could be speculated that a lower bacterial load and diversity in the SENN-DDT strain may also underlie the different responses to antibiotics. The antibiotic treatment may have had a greater effect on reducing an already low bacterial load of poor diversity in this strain, hence the greater effects on longevity and resistance. It must be noted that these observations are based on the analysis of culturable aerobic bacteria, and that the culture of more fastidious organisms, including microaerophilic and anaerobic bacteria may reveal further insights not captured in this study.

It is concluded that the gut bacterial populations of *An. arabiensis* are a crucial determinant of their life histories, significantly affecting adult longevity and the expression of insecticide resistance. The insecticide resistant and susceptible strains used here differ in their gut bacterial milieu, with the resistant strain showing lower bacterial diversity but a predominance of insecticide-degrading species. Although gram-negative bacteria tended to dominate the gut populations, alteration of gram-positive populations induced the most marked effects on the life histories of adult mosquitoes.

## Supplementary information

Supplementary Dataset 1

## Data Availability

All data generated or analysed during this study are included in this published article (and its Supplementary Information files).
