# Supplementary material for: The contribution of gut bacteria to insecticide resistance and the life histories of the major malaria vector Anopheles arabiensis (Diptera: Culicidae)
Source: Sci Rep. 2019 Jun 24;9:9117. doi: 10.1038/s41598-019-45499-z (PMC6591418; doi:10.1038/s41598-019-45499-z)
Supplement: Supplementary file 1 — Supplementary Dataset 1 [file 41598_2019_45499_MOESM1_ESM.pdf]

# The contribution of gut bacteria to insecticide resistance and the life histories of the major malaria vector *Anopheles arabiensis* (Diptera: Culicidae).

Kirsten Barnard[1], Alexander C.S.N. Jeanrenaud [2.3], Basil D. Brooke[2.3], Shüné V. Oliver[2.3*]

1: School of Molecular and Cell Biology, Faculty of Science, University of the Witwatersrand, Johannesburg, South Africa

2: Centre for Emerging Zoonotic and Parasitic Diseases, National Institute for Communicable Diseases of the National Health Laboratory Service, Johannesburg, South Africa

3: Wits Research Institute for Malaria, School of Pathology, Faculty of Health Sciences, University of the Witwatersrand, Johannesburg, South Africa

*Corresponding author: shuneo@nicd.ac.za

## Supplementary Table 1: Bacterial counts of SENN and SENN DDT guts after antibiotic treatment

| | OD600(no. of cells) | | | |
|---|---|---|---|---|
| **Treatment** | **SENN Female** | **SENN Male** | **SENN DDT Female** | **SENN DDT Male** |
| **Control** | 0.813 ($6.5X10^8$) | 0.719($5.75x10^8$) | 0.559 ($4.47x10^8$) | (0.517 ($4.4x10^8$) |
| **Gentamicin** | >0.001* | >0.001* | >0.001* | >0.001* |
| **Vancomycin** | 0.038($3.04x10^7$) | 0.036($2.88x10^7$) | 0.027($2.16x10^7$) | 0.023($1.84x10^7$) |
| **Streptomycin** | 0.015($1.2x10^7$) | 0.013($1.04x10^7$) | 0.011 ($8.8x10^6$) | 0.011 ($8.8x10^6$) |

*= not detectable by our equipment.

**Methodology:**

To confirm the effect of antibiotic treatment 5 individuals of each strain, treatment and sex were removed at the age of 3 days, with only the treated sugar water as a carbon source. The control consisted of the same number of individuals fed on 10% sucrose without antibiotic supplementation. The midguts were removed, homogenised in sterile Phosphate Buffered Saline and the optical density at 600nm (OD600nm) was determined using a Nanodrop™ 2000C spectrophotometer. Number of bacterial per millilitre was determined by calibration to an *E. coli* standard curve. This experiment was replicated 3 times, and the table represents the total average (technical and biological replicates).

**Supplementary table 2: LT50 values of the susceptible SENN strain after blood-delivered antibiotic treatment.** LT50s reported in minutes with standard error in brackets

| | Feeding Treatment | | | | |
|---|---|---|---|---|---|
| | **Control (untreated)** | **Blood only** | **Van(+)** | **Strep(-)** | **Gent(+/-)** |
| **Deltamethrin** | 12.78 (0.68) | 10.02(0.98) | 9.97(0.46) | 10.58(0.48) | 9.78(0.52) |
| **Malathion** | 8.13(0.97) | 9.67(0.35) | 9.05(0.35) | 8.35(0.76) | 9.52(0.62) |

Supplementary Figure 1

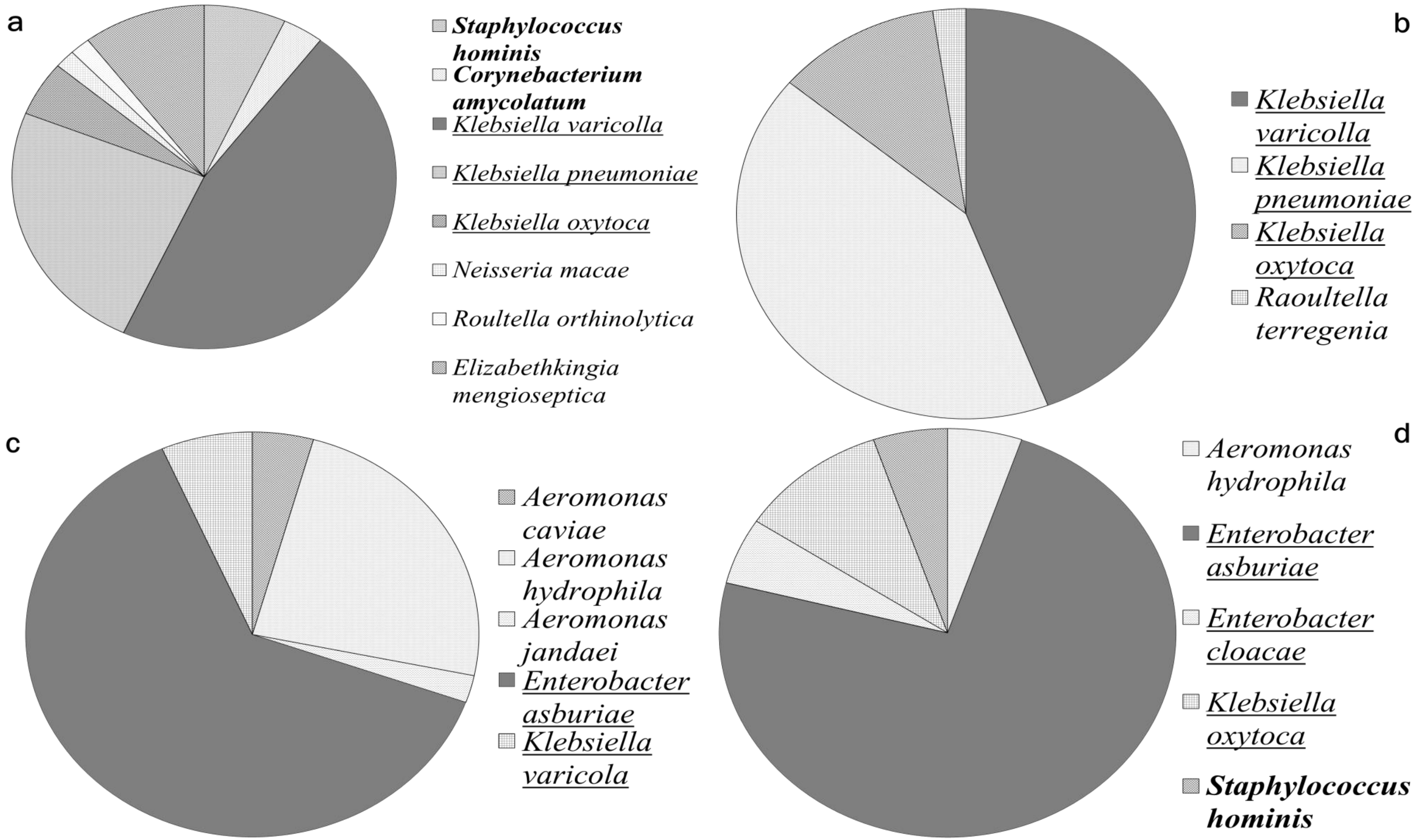

**Supplementary Figure 1: Predominant culturable, aerobic gut bacteria in *Anopheles arabiensis* SENN and SENN-DDT.**

A: Primary species cultured from the gut of fourth instar SENN larvae. B: Primary species cultured from the gut of fourth instar SENN-DDT larvae C: Primary species cultured from the gut of 3-day-old non blood-fed adult SENN females. D: Primary species cultured from the gut of 3 day old non blood-fed adult SENN-DDT females. Gram-positive bacteria are shown in bold. Bacteria previously implicated in bacterial degradation are underlined.

This characterisation is based on the list found in Almeida LG, Moraes LA, Trigo JR, Omoto C, Cônsoli FL. The gut microbiota of insecticide-resistant insects houses insecticide-degrading bacteria: A potential source for biotechnological exploitation. PLoS One. 2017 Mar 30;12(3):e0174754.doi: 10.1371/journal.pone.0174754. eCollection 2017.

**Supplementary Figure 2: Standard WHO bioassay of SENN and SENN DDT**

Standard WHO bioassays as per the WHO guidelines (2016) were performed on 3-day-old, non-blood fed females and males. Four replicates of 25 individuals were exposed to either 5% Malathion (organophosphate) or 0.05% Deltamethrin (pyrethroid). For both Females (Block A) and Males (Block B), both insecticides induced 100% mortality in the SENN strain, while there were survivors in the SENN-DDT strain. SENN-DDT is therefore classed as resistant to both insecticides (Mortality under 98%), while SENN is classed as susceptible (mortality over 98%).

**A**

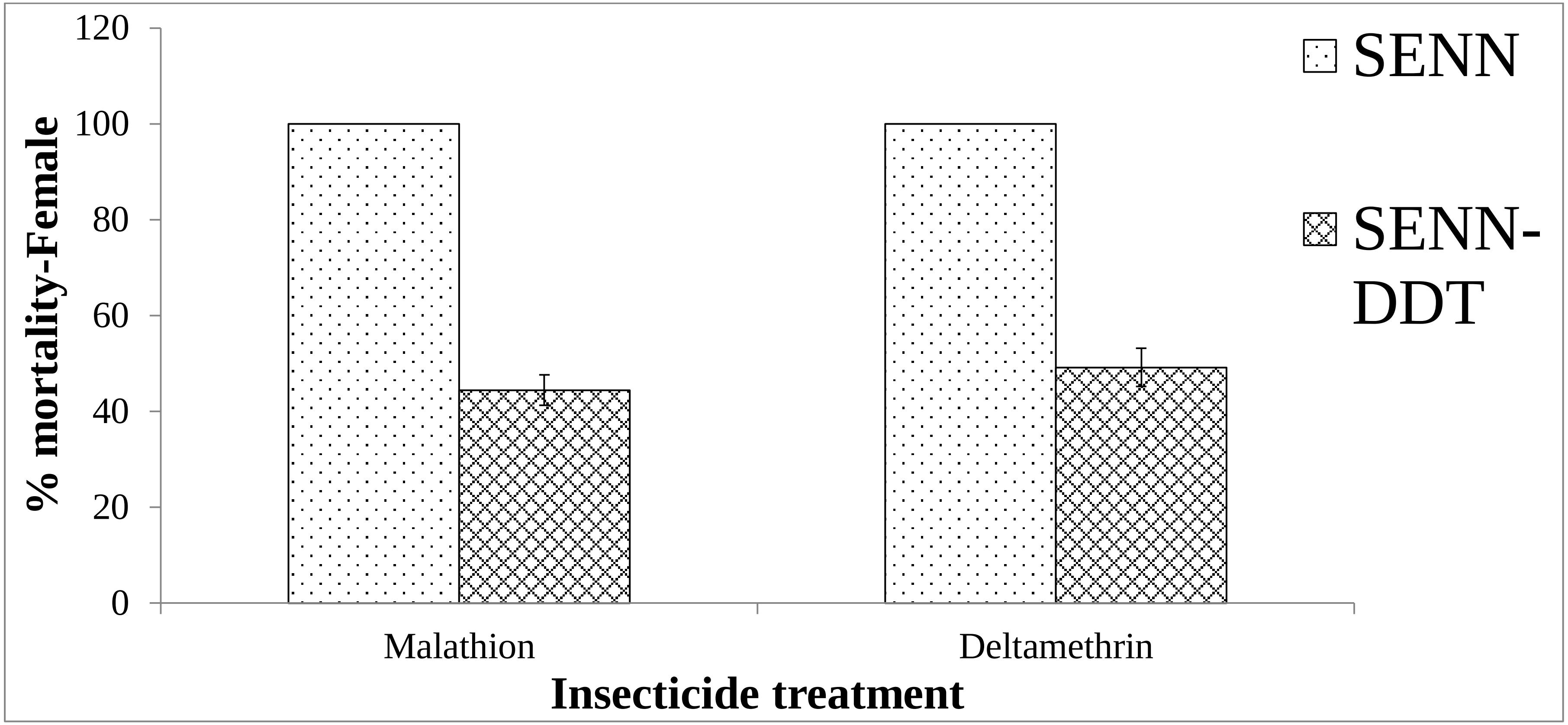

120
100
80
60
40
20
0
% mortality-Female
SENN
SENN-DDT
Malathion
Deltamethrin
Insecticide treatment

**B**

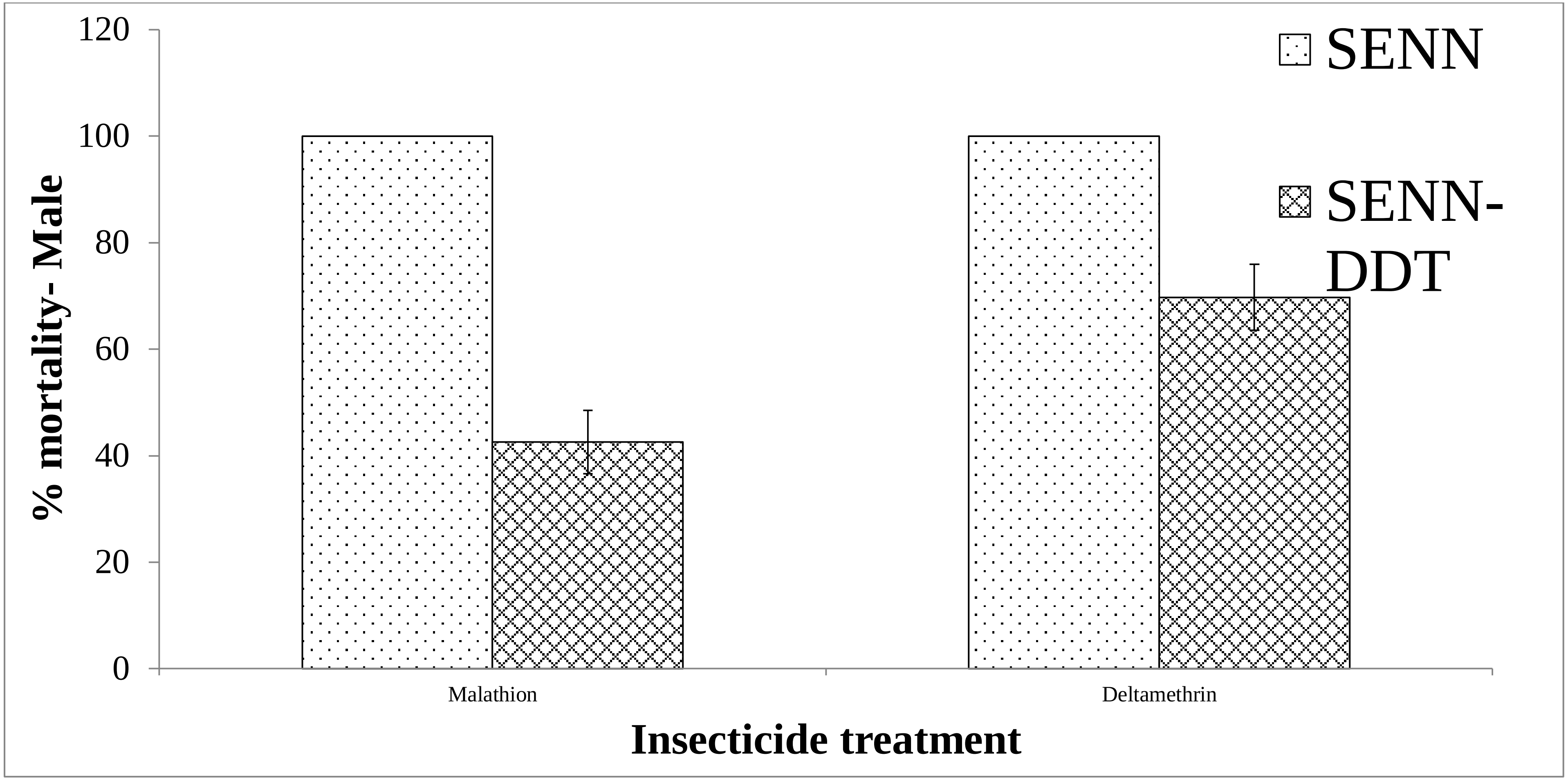

120
100
80
60
40
20
0
% mortality- Male
SENN
SENN-DDT
Malathion
Deltamethrin
Insecticide treatment

**Supplementary table 3A: The effect of erythromycin supplementary on the longevity of SENN-DDT (Time to 50% death-days)**

| | Male | Female |
|---|---|---|
| Control | 24 | 24 |
| Erythromycin | 26 | 25 |

**Supplementary table 3B: The effect of erythromycin supplementary malathion and deltamethrin-induced mortality of SENN-DDT (% mortality)**

| | **Insecticide** | **Control** | **Erythromycin** |
|---|---|---|---|
| **Males** | Malathion | 38.14688 | 42.1632 |
| | Deltamethrin | 33.93954 | 36.9112 |
| **Females** | Malathion | 28.27177 | 30.4956 |
| | Deltamethrin | 34.86569 | 37.4973 |
